# Exploring the effectiveness of the output-based aid voucher program to increase uptake of gender-based violence recovery services in Kenya: A qualitative evaluation

**DOI:** 10.1186/1471-2458-12-426

**Published:** 2012-06-12

**Authors:** Rebecca Njuki, Jerry Okal, Charlotte E Warren, Francis Obare, Timothy Abuya, Lucy Kanya, Chi-Chi Undie, Ben Bellows, Ian Askew

**Affiliations:** 1 Population Council, P.O Box 17643–00500, Nairobi, Kenya

**Keywords:** Voucher program, Gender-based violence recovery services, Health service utilization, Kenya

## Abstract

**Background:**

Few studies in Africa have explored in detail the ability of output-based aid (OBA) voucher programs to increase access to gender-based violence recovery (GBVR) services.

**Methods:**

A qualitative study was conducted in 2010 and involved: (i) in-depth interviews (IDIs) with health managers, service providers, voucher management agency (VMA) managers and (ii) focus group discussions (FGDs) with voucher users, voucher non-users, voucher distributors and opinion leaders drawn from five program sites in Kenya.

**Results:**

The findings showed promising prospects for the uptake of OBA GBVR services among target population. However, a number of factors affect the uptake of the services. These include lack of general awareness of the GBVR services vouchers, lack of understanding of the benefit package, immediate financial needs of survivors, as well as stigma and cultural beliefs that undermine reporting of cases or seeking essential medical services. Moreover, accreditation of only hospitals to offer GBVR services undermines access to the services in rural areas. Poor responsiveness from law enforcement agencies and fear of reprisal from perpetrators also undermine treatment options and access to medical services. Low provider knowledge on GBVR services and lack of supplies also affect effective provision and management of GBVR services.

**Conclusions:**

The above findings suggest that there is a need to build the capacity of health care providers and police officers, strengthen the community strategy component of the OBA program to promote the GBVR services voucher, and conduct widespread community education programs aimed at prevention, ensuring survivors know how and where to access services and addressing stigma and cultural barriers.

## Background

There is increasing global concern regarding gender-based violence (GBV) as a public health issue. Worldwide, the estimated lifetime prevalence of GBV among women is between 15 and 71 percent [1-4]. Estimates from African countries indicate a lifetime prevalence of between 25% and 48% (for example: 48% in Zambia, 47% in Kenya, 34% in Egypt, 30% in Uganda and 25% in South Africa) and an annual prevalence ranging between 10% and 26% [5-7]. Rape and domestic violence are estimated to account for between 5 and 10 percent of healthy years lost by women [8].

Data from Kenya, for example, show that 39 percent of women aged 15–49 years have experienced some form of physical violence from the age of 15 while 45 percent have experienced either physical or sexual violence [9]. Among ever married women aged 15–49 years, 47 percent have experienced physical, sexual or emotional violence from a husband or live-in partner [9]. Over the years, the need to improve access for survivors of GBV services in sub-Saharan Africa (SSA) has received increased attention, given the reported linkage between GBV and reproductive health problems [10-14]. GBV has, for instance, been associated with short birth intervals, increased infant mortality, under nutrition among children of abused mothers, and increased incidence of HIV/AIDS and sexually transmitted infections (STIs) [13,15-19].

The high rates and health effects of GBV documented in SSA have led to discussions and proposals for the integration of GBV services in innovative health financing models such as the “demand-side” health financing (DSF) or “output-based aid” (OBA). The goal of these health care financing models is to increase access to and uptake of key services by offering sufficient subsidies and resources to enable the user to purchase the service, preferably by being able to choose a provider from among a number of alternatives [20,21].

In OBA programs, a voucher management agency (VMA) distributes or sells vouchers at a subsidized price to clients, who purchase a voucher for a specific service. OBA programs provide incentives to clients and healthcare workers and subsidize specific health care packages based on the provision of care with pre-defined quality standards and pre-determined outputs [22,23] with the goals of improving service quality, stimulating client use of selected services, targeting services among high-priority populations (such as the poor or underserved), and containing costs [24-28]. In some programs there is a supervisory or regulatory body that meets periodically to oversee their functioning. The structure of the OBA programs is such that it identifies and invites individual or networked service providers (public, non-profit or for-profit) to assess their suitability to participate. Those agreeing to participate can only do so if they can demonstrate service provision at a specified standard of quality of care; they are then accredited to participate subject to regular review. Usually a number of providers are accredited to create competition and give consumers choice. When the client needs the services, s/he then redeems the voucher for the specified service at one of the accredited facilities. The provider is then reimbursed service cost or paid an incentive upon submission of a claim and supporting evidence to the VMA.

In Kenya, the reproductive health voucher program was launched in 2005 with an overall aim of increasing skilled birth attendance for women seeking maternal and newborn services, increasing utilization of long-term family planning methods, and increasing the uptake of gender-based violence recovery (GBVR) services for both men and women. The program contracts both public and private facilities to provide a comprehensive reproductive health service package [29]. The OBA program is currently being implemented in five sites across five provinces in Kenya namely: Kisumu, Kitui, Kiambu, and Kilifi districts, and Korogocho and Viwandani informal settlements in Nairobi. In total 10 hospitals were accredited to provide GBVR services.

The GBVR services vouchers are made freely available to women at the facility; there is no community-based distribution and no specific selection criteria for identifying eligible clients, such as a poverty grading tool used to identify beneficiaries for family planning and safe motherhood services [20]. Each accredited facility has a stack of vouchers which is used to submit a claim after services are rendered to clients. The GBVR services vouchers provide access to a wide range of services, including: (i) a medical examination, treatment and management of injuries, hospitalization and accommodation, laboratory testing and X-rays, pregnancy prevention services and HIV post exposure prophylaxis (PEP), (ii) counseling services specifically consisting of psychological care, trauma counseling, crises management, HIV pre-and post-test counseling, and adherence counseling, and (iii) links to support groups which provide legal aid, monthly group therapy sessions for survivors of rape, information and referral for long-term shelters, and help for survivors in liaising with social services departments and private sector support for medical services.

Although the voucher program has been successful in increasing skilled birth attendance, uptake of long-acting family planning methods, and reducing out-of-pocket expenditure [30,31], there is no evidence to date regarding the effectiveness of the voucher approach with respect to improving access to GBVR services. This paper therefore explores the extent to which the Kenya OBA GBVR services are viewed as effective from the perspectives of different actors including any perceived barriers to the use of GBVR services.

## Methods

The paper draws on qualitative data including 97 in-depth interviews (IDIs) and 27 focus group discussions (FGDs). A total of 69 IDIs were conducted with health managers and service providers, three with VMA managers, and 25 with government administration officers at the district level. The FGDs were, on the other hand, held with female voucher users and non-users, voucher distributors and opinion leaders such as local village elders, chiefs and community health workers drawn from the five program sites. Qualitative data provided insight on the utilization, perceptions of and experiences with the GBVR services vouchers as well as on awareness, understanding and attitudes towards the voucher program. The FGDs consisted of between six and eight participants with discussions lasting one to two hours. The IDIs and FGDs were tape recorded and transcribed into a Microsoft Word file. The transcribed texts were then transferred to NVIVO 8 analysis software. Data analysis was done by two researchers. Following coding, a full list of themes was available for categorization within a hierarchical framework of main and sub-themes. The thematic framework was then systematically applied to all of the interview transcripts. We looked for patterns and associations of the themes and compared and contrasted within and between the different actors with specific focus on awareness and understanding of GBV vouchers and services, stigma and access barriers, opportunities and challenges for GBV program functioning and barriers for legal redress.

Ethical approval for conducting the study was granted by the Population Council Institutional Review Board and the Kenya Medical Research Institute Ethical Review Committee [32].

## Results

### Awareness and understanding of GBVR vouchers and services

Qualitative findings demonstrated a low of awareness of the GBVR voucher and lack of understanding of the benefit package offered. In particular, providers and health managers exhibited a poor understanding of what the benefit package entailed. There were also conflicting statements on the procedures to be followed while providing medical treatment to survivors. For example, some providers stated that the law requires clients to report to the police first and obtain the police medical examination report (also known as the “P3” form) in order to get treatment. There also appears to be lack of clarity on who is mandated to fill in the P3 form. This led to calls for additional training and clarification on procedures to follow in offering GBVR services.

"They need to include other things – for example, train on counseling on how to handle rape care, which has been challenging. Also, train on the P3 forms [post-rape forms], which are not well known….also train on long-term family planning methods and more nurses should be trained on emergency contraception because they are few (IDI, service provider)."

In addition, many participants commented that the GBVR services voucher is little known by beneficiary communities and those with some level of knowledge lacked adequate information on the service package and where the accredited facilities were located. In particular, the majority of women (both those who had used other vouchers and those who had not) indicated that they did not know the GBVR services sites or they had difficulties accessing the accredited facilities. A participant, for instance, noted:

"I know of the safe motherhood and family planning vouchers. This one you are asking I have never heard about it (FGD, voucher user)."

"We have not been given information adequately by the voucher distributors; they should put more effort so that the vouchers are brought near. Some do not know about the vouchers; they are not enlightened. Educate people because many have not heard about the GBV voucher you are asking [us about]. Educate them in the barazas [weekly meetings held by chiefs and assistant chiefs] at the chief’s place by bringing educators (FGD, voucher user)."

### Stigma and access barriers

Although providers acknowledged that the GBVR services voucher has expanded access to services among some segments of the poor, it was widely believed by many community members that GBV is a minor offence or “family matter” that can be easily dealt with. Many families withhold information on sexual GBV-related cases with the intent to protect the family name. Similar views were held by government officers, VMA managers and members of the community who observed that deeply-rooted stigma hampers access to GBVR services.

"… When we talk about gender-based violence in our area, people still don’t understand it very [well], and people relate it to the family. What if I go tell my husband about it? What will happen to my marriage? How will the people in the community perceive me? What about my family members, how will they perceive me? That is why you see the uptake is low. People are trying to safeguard their families, no matter how bad things are [perceived in the community] (IDI, service provider)."

"I think it (GBV) is much stigmatized. If we can de-stigmatize that system then it would be better. People will access it. Most of these rape cases or violence occurs at home. It’s by people who know you within the families, so when it is taking place within the family, these are the same people who are supposed to take you to hospital. Do you want to expose that your father has raped you or your brother or your cousin? So most of them take place within the family. Even if it is known, it is discussed within the family and that matter is closed (IDI, District Officer)."

"The gender violence voucher is a bit tricky because not many mothers will come in and say I was raped because of the stigma (IDI, VMA Manager)."

"I think also…there is fear to go to health facility. Fear of being known that she had been raped or something like that, so she keeps it to herself and can’t tell anybody. So you find these cases are not reported and they can’t get the cards for subsequent services (FGD, voucher user)."

Discussions with voucher distributors also revealed community members’ perceptions that there are hidden costs in the voucher program and a reluctance to use free services. There is evidence in the narratives that community members were skeptical about the GBVR services voucher being entirely free. As one voucher distributor explained:

"I believe it is not used because it is only found in the facility [as the distribution point]… I, as a voucher distributor based in the community, know that the locals believe I have two vouchers with me; none is free, so how can the GBV voucher be free? I think maybe they go to the facilities and are not aware the GBVR services are free. Before, GBV costs were around 1000 shillings, now if they don’t know about the information they think they will be charged when they go there (FGD, voucher distributor)."

To address some of the barriers to GBVR services mentioned, voucher distributors and the VMA field managers recommended that community sensitizations be intensified by working closely with local authorities and administration to create general awareness of the GBVR services vouchers and information on where to obtain services. Additionally, voucher distributors felt that they should be allowed to hand out GBVR services vouchers in the community to dispel the belief that they are for sale.

### Opportunities and challenges for GBV voucher program functioning

Poor understanding of GBVR service voucher program, lack of essential commodities and the dearth of trained personnel mean that most facilities do not have the capacity to adequately provide comprehensive GBVR services. The situation is dire for rural-based facilities that face perennial shortages of supplies and understaffing. For instance, health providers at a recently upgraded sub-district hospital (from health centre status) indicated that although they were endorsed to offer GBVR services, they lacked essential supplies and personnel to support the services. There was also a common perception that the referral service was rarely used by patients because of the long distances covered and time taken to access services.

"We normally refer to the District Hospital though the [GBVR] vouchers have been given to the sub district hospitals too. But the laboratory facilities are not in the sub district facilities so the best thing is they should equip the labs in the sub district hospitals (IDI, service provider)."

"Also this person may come and most of the hospitals of this category may not be able to handle those cases of sexual violence. They may not have the ARVs [antiretroviral drugs] to give; they may not have the tests to do. So you may have come, you’ve been raped and then you are referred to another institution so that system takes time and you feel it’s not really worth it. Therefore most of them give up on the way (IDI, service provider)."

Many community distributors explained that marketing GBVR services vouchers was a difficult task given the longstanding social and cultural taboos observed in most communities. Moreover, the poor understanding of the voucher was a result of the single source marketing strategy used to promote the services (only voucher distributors were used to sensitize the community about these services). According to the distributors, multiple sensitization strategies are required to break the barriers. Many felt that over reliance in them or their peers to pass on critical GBVR services information is not enough to break some of the barriers that communities still hold. To counter these barriers the distributors noted that important additional information should be availed to community members.

"I support that a strong road show should be done about the gender violence voucher so that it can be in public domain. We also as distributors should be given the GBV voucher so that when we are in the community, where they know our telephone numbers they can inform us about it. And when she contacts the distributor, you will tell the patient to go to the accredited hospital and confirm that there will be no payment as she will be afraid that now I’m going to be charged because they are normally afraid about being charged (FGD, voucher distributor)."

"It (GBV voucher) is not being used due to the lack of enough information. I can say that information on where the voucher is given and where one can get and use the voucher is not known in this community especially in the interior areas (FGD, voucher distributor)."

Our findings also suggest that the utilization of the GBVR services voucher is to a great extent affected by the perceived quality of care provided at various facilities. Women and voucher distributors noted that some facilities are not entirely trusted by the community. Many distributors were of the view that community members distrust staff’s ability to maintain client confidentiality and offer quality services. It was felt that some facilities score poorly in service provision leading to reluctance to visit such centers. Focus group discussions with community opinion leaders reinforced concerns that facilities lacked designated units to attend to sexual assault survivors. In most cases, survivors had to follow the same procedures used by regular patients.

"I believe it (GBV voucher) is not used because it is distributed in the facility. This facility treats them badly, even rape cases, and they do not have faith in them (FGD, voucher distributors)."

"There is a problem with the gender violence desk when you seek services for sexual assault because you will go through the same process a sick person goes through (FGD, community health workers)."

Service providers noted that some accredited facilities, especially public facilities, lack the required equipment and supplies. Some clients apparently seek care, but experience delays in receiving appropriate treatment due to frequent stock-outs. The participants also reported that not all providers, especially those in rural facilities, have the necessary skills to handle GBV cases. As a result such facilities often end up referring clients even though a majority of the population are poor and cannot afford the transportation costs.

"…even I, as facility manager, don’t know for example if they (GBV survivors) did come to my place what am I supposed to do? I just do my first aid and just check to see that they are physically okay. Then for the further management I just refer them to facility xxxxxx. In between, I don’t know what is happening (IDI, facility manager)."

### Challenges with seeking legal redress

The most common reason stated as preventing GBV survivors from seeking justice was the inability of the criminal justice system to apprehend and prosecute the perpetrators. According to respondents some individuals in position of power such as village elders or the provincial administration collude with suspects to drop the charges, ensure that the cases are concluded in favor of the suspects, or that the cases take longer than necessary before a ruling is passed. In addition, many respondents felt that the law enforcement agencies are not well trained to handle sexual assault cases. The significance of seeking legal redress and whether individuals ever get justice emerged as a sub-theme within this discussion. The subject drew various reactions, for example:

"If you take a sexual case to the police, you know the way the police handle such cases [the survivors] are really treated so badly. They don’t feel like going back or following up that matter, so I think there should be good customer care (IDI, service provider)."

"… ..you know the community fears breach of confidentiality, so we should inform them [survivors] that when they seek medical assistance the matter would be confidential. If the report goes directly to the police stations or the elders, the matter would not be confidential, so the best thing is to seek medical treatment without going through the police and elders (FGD, voucher distributor)."

In summary, inadequacy of the investigative process and legal representation was cited as undermining the due process of the law. It was felt that at times the police lacked the ability to successfully investigate and see the cases to the prosecution stage. Lack of resources on the part of the community to afford legal representation is also a deterrent. There were also ambiguities on which procedures to follow; it is not clear to providers whether one should seek treatment first or report to the police.

## Discussion

The views of the different actors provided important insights on the perceptions and barriers to implementation and factors affecting utilization of GBVR services subsidized by the voucher program in Kenya. It was clear from the narratives that all the actors had limited knowledge of GBVR vouchers and most recommended improving community engagement and sensitization about the voucher program. In addition, service providers and voucher distributors indicated that community members lacked assurance that the GBVR services voucher provides free services. Lack of basic information on the GBVR services prevents many survivors from seeking care or taking up other support services from medical and legal institutions.

Our findings illustrate poor community knowledge of GBVR services vouchers, the benefit package and the need for treatment. This points out to a need to improve community awareness and engagement with the GBVR services voucher program. The active participation of the local community in planning, implementing, and monitoring interventions is a crucial factor in successful implementation of any program. Recent studies have reported that bottom up designs that emphasize community-driven interventions are more successful compared to those initiated using a top down approach [33,34]. There is an opportunity for the program to explore partnering with community organizations, women’s groups and local leaders to improve community awareness of the GBVR services voucher.

Cultural factors and stigma emerged as key barriers to the uptake of GBVR services. For instance, how to deal with cases of defilement and rape by close relatives has been noted as a key deterrent to the utilization of GBVR services. The fact that community norms and beliefs have played a role in protecting perpetrators of sexual assault cannot be underestimated. This is consistent with other studies that show cultural factors as the single most important obstacle for low utilization of health services even when a transport system is in place [35,36]. Non-legal, community sanctioned mechanisms of addressing sexual assaults such as compensation of the family at an agreed rate or involving provincial administration officers are seen as a substitute to medical treatment which undermines utilization of GBVR services. Perceived lack of privacy and confidentiality in the entire process of seeking forensic evidence and completing P3 forms to seek medical treatment or start the legal redress process is viewed as part of disclosing information about the perpetrator. These perceived fears should be addressed to improve uptake of GBVR services through education of providers and police officers on the need for maintaining confidentiality for GBV survivors. Further, the treatment the survivors get at the first point of contact is critical but overlooked, and survivors are often humiliated. For example, police officers are reported to handle survivors poorly and consequently the majority of them give up seeking GBVR services at this point. The program should perhaps explore partnering with police officers and local community-sanctioned forums to encourage referral to facilities and access to justice.

As already noted, community referral and transport system is a key challenge in the utilization of GBVR services. A poor transport network and lack of money to pay for transport costs have been associated with delays in reaching a health facility and low utilization of the GBVR services voucher. It also has implications for the 72-hour ‘window of opportunity’ for forensic examination and medical management of survivors. The distribution point of the GBVR services voucher at the facility appears to pose a challenge especially for rural residents given that all facilities accredited to offer GBVR services are hospitals mainly located in urban areas. In addition, the survivors do not have an adequate choice of facilities from where to seek the services due to the sparse distribution of the accredited providers. The inability of the existing distribution system to effectively reach rural areas suggests the need to build capacity for lower level facilities to provide GBVR services. Whereas the voucher program provides transport and referrals for only life-threatening complications, most gender-based violence cases are not perceived as life threatening, however they do require specialized care within a specific time period. In cases of referral, failure to afford the costs means that the client will not seek the required care. Although the OBA program finances all the facility-based costs for gender-based recovery services, it does not cover transportation costs and this presents an important missed opportunity. Transportation costs within the voucher program have been noted as a major contributing factor to low utilization of reproductive health services [22,27]. Putting in place mechanisms for transport reimbursement could potentially improve GBVR services uptake within the voucher program.

Although one aim of the voucher program is to encourage provision of quality care, this is being undermined by facilities with service providers who are not competent to provide GBVR services, and a lack of supplies and equipment. Furthermore, many service providers do not understand the GBVR services benefit package. To achieve program objectives, quality care should be improved through capacity building of providers to offer GBVR services. There is also need for strengthening the health system. The program has an opportunity for working closely with local community health workers and midwives to provide basic treatment and speedy referrals to improve the number of GBV survivors receiving critical care within 72 hours. In addition, it appears that poor treatment of survivors by providers is a deterrent to utilization of GBVR services. For example, the majority of women and community opinion leaders maintained that in the community, a previous bad experience with a health care provider influences the choice of whether a person will seek facility treatment or not. This is consistent with findings from other studies that show that the poor treatment of clients affects health care service utilization [35].

There is a need to address the confusion among providers regarding the process required in terms of timing to seek medical management of rape and sexual violence. In Kenya, rape survivors are encouraged to first seek medical attention and immediately after report to the police. However, if survivors report to the police first, they should go to the health facility immediately after. Providers need to be sensitized that contrary to what some of them think, the P3 form does not have to be completed immediately; this can be done after the medical evaluation is complete [37]. A post-rape care form (PRC1) allows for the history taking, documentation and examination. The PRC1 form is filled by a trained clinician at health facilities to provide clinical notes that facilitate the filling of the P3 form by ensuring that all the relevant details are available. The police provide the P3 form that should be completed by an authorized health worker based on the clinical notes found in PCR1.

Overall our findings suggest a need for inter-sectorial collaboration to develop new linkages and strengthen existing ones in order to improve the uptake of GBVR services. It appears that the scarcity of referral linkages between different levels of facilities result in missed opportunities for prompt treatment. The 72-hour ‘window of opportunity’ for forensic examination and medical management requires speedy and efficient referrals. To reduce delays in seeking timely treatment, there is need to address the general confusion over protocols and procedures among different sets of players, develop effective referral mechanisms, and strengthen collaboration among facilities of different sectors.

## Conclusions

Overall, the OBA voucher program has been shown to increase skilled birth attendance and uptake of long-term family planning methods. It is therefore imperative that barriers surrounding the uptake of the GBVR services be addressed in order to increase demand for the services. As highlighted in this paper, various factors affect the successful implementation of the GBVR services voucher program. These include access to accredited providers, difficulty in seeking justice, lack of understanding of the GBVR services voucher by target populations, perceived lack of privacy and confidentiality, the requirement to collect forensic evidence and poor handling of survivors by health care providers and the police. These findings suggest that there is a need to build the capacity of health care providers and police officers, strengthen the community strategy component of the OBA program to promote the GBVR services voucher, and conduct widespread community education programs aimed at prevention, ensuring survivors know how and where to access services and addressing stigma and cultural barriers.

## Limitations of the study

In the study setting, it was difficult to recruit survivors of GBV and therefore the findings are based on general community and provider perceptions, knowledge of GBV and the GBVR services voucher. The researchers could not distinguish survivors among the study participants. In-depth interviews with the survivors could have possibly given a better perspective of the effectiveness of the OBA voucher program regarding improving access to and uptake of GBVR services. Another limitation of the study stems from the fact that participants in the IDIs and FGDs were not randomly selected, which may affect the generalization of the findings to the populations covered by the voucher program. However, the selection of participants was aimed at capturing the views of actors representing various groups of people in the community (voucher program managers, voucher distributors, service providers, the local administration, and the intended beneficiaries) with the hope that consistent themes across the groups would suggest that the issues are generalizable to the community. There is also the potential for subjectivity in the analysis and interpretation of the findings, which was addressed by having two researchers conduct the analysis and compare findings.

## Abbreviations

DSF, Demand side financing; FGD, Focus group discussions; GBV, Gender based violence; GBVR, Gender-based violence recovery; IDI, In-depth interviews; OBA, Output-Based Aid; PEP, Post exposure prophylaxis; PCR1, Post rape care form; STI, Sexually transmitted infections; SSA, Sub Saharan Africa; VMA, Voucher management agency.

## Competing interests

The authors declare no competing interest.

## Authors’ contribution

RN: Involved in data collection, data analysis, interpretation, drafting, re-organizing and overall revision of the manuscript, JO: Involved in data analysis, interpretation of the data, re-organizing and revision of the manuscript, CEW: Involved in the conception of the design of the study and revision of the manuscript, FO: Involved in data collection, and the revision of the manuscript, TA: Involved in data collection, and the revision of the manuscript, LK: Involved in the revision of the manuscript, CU: Involved in the revision of the manuscript, BB: Involved in the conception of the design of the study and revision of the manuscript, IA: involved in the conception of the overall study design. All authors read and approve the final manuscript.

## Pre-publication history

The pre-publication history for this paper can be accessed here:

http://www.biomedcentral.com/1471-2458/12/426/prepub
